# Integrative Epigenomic and Transcriptomic Profiling Define Malignancy- and Cluster-Specific Signatures in Pheochromocytomas and Paragangliomas

**DOI:** 10.3390/cells15020198

**Published:** 2026-01-20

**Authors:** Mouna Tabebi, Małgorzata Łysiak, Oliver Gimm, Peter Söderkvist

**Affiliations:** 1 Department of Biomedical and Clinical Sciences (BKV), Linköping University, 58183 Linköping, Sweden; malgorzata.lyziak@liu.se (M.Ł.); oliver.gimm@liu.se (O.G.); peter.soderkvist@liu.se (P.S.); 2 Clinical Genomics Linköping, Science for Life Laboratory, Linköping University, 58183 Linköping, Sweden; 3 Clinical Department of Surgery Linköping, Region Östergötland, 58183 Linköping, Sweden

**Keywords:** pheochromocytoma/paraganglioma, methylation, transcriptomic, TCGA

## Abstract

Pheochromocytomas and paragangliomas (PPGLs) are rare neuroendocrine tumors primarily involving the adrenal medulla and its associated paraganglia, with heterogeneous clinical behavior and complex molecular drivers. This study aimed to characterize DNA methylation and gene expression patterns in PPGLs to understand the molecular differences between tumor subtypes and malignancy. We performed an integrative analysis of DNA methylation (Illumina EPIC 850K) and gene expression profiles (Affymetrix microarrays) in 24 PPGLs, comparing these with The Cancer Genome Atlas (TCGA) data, to delineate cluster- and malignancy-specific epigenetic patterns. Comparison between pseudohypoxic Cluster I and kinase-signaling Cluster II tumors revealed 13 differentially methylated CpG sites, with a specific CpG within *DSCAML1* showing hypermethylation in Cluster II accompanied by increased expression, suggesting context-dependent gene body methylation effects. Benign versus malignant comparisons identified 101 differentially methylated CpGs, including hypermethylated CpG in *BAIAP2L1* and hypomethylated CpG in *SHANK1* in malignant tumors. Pathway enrichment of differentially methylated genes revealed alterations in Notch signaling, adherens junctions, cytoskeletal regulation, and intracellular transport. Gene expression analysis demonstrated partial overlap between clusters, with malignant tumors exhibiting distinct transcriptional profiles involving RNA processing, metabolism, and adhesion pathways. Correlation between methylation and expression was generally limited, emphasizing that methylation-dependent gene regulation is a locus-specific and context-dependent regulation. These findings illustrate a complex interplay between epigenetic modifications and transcriptional programs in PPGLs, enhancing our understanding of molecular heterogeneity and tumor classification, and identifying candidate biomarkers and therapeutic targets for malignant progression.

## 1. Introduction

Pheochromocytomas (PCCs) and paragangliomas (PGLs), collectively known as PPGLs, are rare neuroendocrine tumors arising from chromaffin cells of the adrenal medulla and extra-adrenal paraganglia, respectively. First described over a century ago, when their distinctive catecholamine-producing properties were recognized, PPGLs have since intrigued clinicians and researchers due to their clinical heterogeneity, variable behavior, and complex underlying biology. Early diagnostic challenges and limited therapeutic options fueled decades of research, culminating in an evolving understanding of the molecular drivers that underpin these enigmatic tumors [1].

The advent of molecular genetics revolutionized PPGL research, revealing that these tumors are among the most genetically diverse human neoplasms. Germline mutations account for approximately 30–40% of PPGL cases, a remarkable figure that emphasizes their strong hereditary component [2]. Central to this discovery was the identification of mutations in the succinate dehydrogenase (SDH) complex genes (*SDHA*, *SDHB*, *SDHC*, and *SDHD*), which encode critical mitochondrial enzymes of the tricarboxylic acid (TCA) cycle and electron transport chain. Loss of SDH function leads to the accumulation of succinate, an oncometabolite that inhibits α-ketoglutarate-dependent dioxygenases, including prolyl hydroxylases responsible for degrading hypoxia-inducible factors (HIFs). This metabolic derangement induces a pseudohypoxic state that drives angiogenesis, metabolic reprogramming, epigenomic dysregulation, and tumorigenesis [3,4].

Parallel discoveries unveiled mutations in other susceptibility genes, such as *VHL*, *RET*, *NF1*, *TMEM127*, and *MAX*, with each contributing to distinct molecular pathways including oxygen sensing, kinase signaling, and cellular proliferation [5]. Furthermore, somatic mutations in genes like *EPAS1* (encoding HIF-2α) and *ATRX* have been implicated in tumor development and aggressiveness, broadening the genetic landscape of PPGLs and highlighting the heterogeneity even within tumor subtypes [6,7].

More recently, a molecular taxonomy of PPGLs has emerged, commonly categorized into three major clusters: *Cluster I* (“pseudohypoxia”), driven by alterations in genes (e.g., *SDHx*, *FH*, *VHL*, *EPAS1*) that activate the TCA cycle and the hypoxia pathway [8,9]; *Cluster II* (“kinase signaling”), driven by mutations in genes (e.g., *RET*, *NF1*, *MAX*, *TMEM127*) that activate the receptor tyrosine kinase/RAS pathway [8,10]; and *Cluster III* (“WNT signaling”) which is rarer, and characterized by *MAML3* fusions and WNT pathway alterations [11,12]. This structured classification has multiple advantages: it links the genotype to distinct transcriptomic/epigenomic signatures, correlates with biochemical phenotype (e.g., catecholamine secretion pattern), and stratifies the risk of metastatic behavior [6,8].

The interplay between genetics and epigenetics has emerged as a critical area of investigation, revealing that metabolic alterations reverberate through the epigenome to influence tumor behavior. Succinate accumulation in SDH-mutated tumors leads to the inhibition of TET family enzymes, resulting in widespread DNA hypermethylation known as the cytosine–phosphate–guanine (CpG) island methylator phenotype (CIMP). This epigenetic signature silences tumor-suppressor genes and reinforces oncogenic pathways, representing a hallmark of pseudohypoxic PPGLs [3]. Additionally, dysregulation of histone modifications and non-coding RNAs further remodel chromatin architecture and gene expression, contributing to tumor progression and heterogeneity [13].

Despite major advances in molecular classification, the extent to which epigenetic and transcriptional programs interact to define PPGL subtype and malignant potential remains incompletely understood. Therefore, in this study, we aimed to (1) compare DNA methylation expression profile between Cluster I and Cluster II PPGLs, (2) identify methylation signature associated with benign versus malignant tumors, and (3) integrate methylation and expression data to reveal locus-specific and pathway-level regulatory patterns relevant to tumor behavior. Integrative multi-omic analyses have refined PPGL classification by linking genetic, epigenomic, and transcriptomic profiles. This approach not only enhances diagnostic precision but also informs therapeutic strategies, creating opportunities for interventions that exploit metabolic vulnerabilities or target epigenetic regulators. By mapping the full spectrum of PPGL heterogeneity, these integrative analyses translate molecular insights into precision clinical strategies, bridging mechanistic understanding and individualized patient care.

## 2. Materials and Methods

### 2.1. Cohort

Twenty-four verified PPGLs were included in this study and were collected at the University Hospital in Linköping, Sweden. Samples were obtained with informed patient consent and with approval from the responsible ethic committee in Sweden (Dnr 2010/40-31, Dnr 2015/175-32, Dnr 2023-04838-02). These samples constitute the experimental (in vitro) component of the study and were subjected to DNA methylation and gene expression analyses to investigate cluster- and malignancy-specific molecular patterns. Tumor tissues were processed following Standard Operating Procedures: resection specimens were placed in labeled cryovials and snap-frozen in liquid nitrogen within 15 min of resection. Peripheral blood samples were also collected from the same patients and used for germline or somatic gene variant (mutation) analysis. Tumors were classified as benign or malignant following the Endocrine Society Clinical Guidelines Subcommittee (CGS) criteria [1]. All cases were histologically confirmed as PPGLs using World Health Organization (WHO) criteria and the cohort characteristics are outlined in Supplementary Table S1.

The mean age of diagnosis was 55.9 years (range 13–76.2 years) and the gender ratio (female/male) was 1.09. The tumors were previously studied for mutations in known driver genes using a custom panel for NGS (Supplementary Table S1).

### 2.2. DNA Extraction and Methylation Array Analysis

DNA was extracted from tissue and blood samples using the Maxwell 16 Tissue DNA Purification Kit (Promega, Madison, WI, USA) and Blood DNA Purification kit (Promega), respectively, following the manufacturer’s recommendations. The extracted DNA was measured fluometrically, and 250 ng was Bisulfite-converted using the EZ DNA Methylation kit (ZYMO Research, Irvine, CA, USA). The final product was analyzed using Illumina Infinium MethylationEPIC v1.0 legacy Bead Chips (850K) (Illumina Inc., San Diego, CA, USA). The array has probes for 866,836 CpG-sites, located in the promotors and gene bodies of ~20,000 protein-coding genes spread across the genome.

### 2.3. RNA Extraction and Microarray Gene Expression Analysis

Total RNA was extracted with the RNeasy Minikit (Qiagen, Hilden, Germany), quantified using a NanoDrop spectrophotometer (ThermoFisher, Waltham, MA, USA), and quality-checked on an Agilent Bioanalyzer (Agilent, Santa Clara, CA, USA); samples with RIN ≥ 7 were included. Sense-strand cDNA was prepared with the Ambion WT Expression Kit (Thermo Fisher Scientific, Austin, TX, USA), then fragmented, labeled, and hybridized to Affymetrix Human Gene 1.0 ST microarrays (Thermo Fisher Scientific, Santa Clara, CA, USA), covering 28,869 annotated genes with a median of 26 probes per gene, following manufacturers’ protocols. Arrays were scanned using a GeneChip Scanner 3000 7G (Thermo Fisher Scientific, Santa Clara, CA, USA), and data were processed with Transcriptome Analysis Console (TAC) v4.0. Normalization used robust multi-array average (RMA) method, and differentially expressed genes (DEG) were defined as q-value < 0.05 and a fold change >2 or <−2.

### 2.4. TCGA PPGL Methylation and Transcriptomic Data Analysis

Complementing our experimental analyses, we performed an in silico re-analysis of the publicly available TCGA-PCPG data (*n* = 173), obtained from the GDC data portal (https://portal.gdc.cancer.gov/projects/TCGA-PCPG accessed on 28 November 2025) [2], to validate cluster- and malignancy-associated methylation and transcriptional patterns. DNA methylation and RNA-seq data were processed using standard pipelines, as described below. Methylation data from the Illumina HumanMethylation450 BeadChip (Illumina Inc., San Diego, CA, USA) were processed in R (v4.4) using the minfi (v1.40.0) and ChAMP (v2.34.0) packages, with low-quality and cross-reactive probes removed and BMIQ normalization applied. Differential methylation analysis between groups was performed using limma (FDR < 0.05, Δβ ≥ 0.2). Corresponding RNA-seq data were normalized and analyzed using DESeq2 to identify differentially expressed genes (FDR < 0.05, fold change >2 or <−2.).

An integration of methylation and expression profiles was conducted to assess the correlations between methylation and gene expression. The visualization of significant features was performed using the ComplexHeatmap (v3.19).

### 2.5. Further Data Processing and Statistics

The IDAT files (Illumina data files) from EPIC arrays were analyzed using R (v4.4) and bioconductor packages (v3.19), Chip Analysis Methylation Pipeline (ChAMP) analysis package (v2.34.0) [14].

CpGs with detection *p*-value > 0.01, SNP CpGs, unbound and multi-hit CpGs, and CpGs from XY chromosome were filtered. Normalized β-values were obtained using beta-mixture quantile normalization (BMIQ).

To reduce the batch effect in relation to biological variation in the data matrix, deconvolution (singular value decomposition, SVD) was performed on the normalized data using runCombat function and corrected against the confounding factors (e.g., Gender, Slides).

The differential methylation analysis was conducted using linear modeling (lmFit) and eBayes algorithm. Significant differentially methylated CpGs (DMCs) had Benjamini–Hochberg (BH)-corrected *p*-values (*p*-value BH) < 0.05.

CpG were categorized as hypomethylated (β ≤ 0.2), hypermethylated (β ≥ 0.8), or partially methylated (0.2 < β < 0.8), thresholds that align with common practice in epigenetics studies. Pathway enrichment analysis was performed using KEGG annotations. The Manhattan plot was generated using qqman package (v0.1.9).

DNA methylation age (mAge) was calculated using the Horvath pan-tissue clock [15], which estimates biological age from methylation at 353 CpG sites. Preprocessed beta values from the Illumina Infinium EPIC (850K) array were used as input. Age acceleration was defined as the residual from a linear regression of mAge on chronological age.

The correlation between DNA methylation (β-values) and gene expression (RMA-normalized microarray values) was evaluated using Pearson’s correlation test. Correlation coefficients (*R*) and corresponding *p*-values were calculated, and significant correlations were defined as |*R*| ≥ 0.7 with *p* < 0.05.

## 3. Results

### 3.1. Methylation Analysis

**Figure 1:**
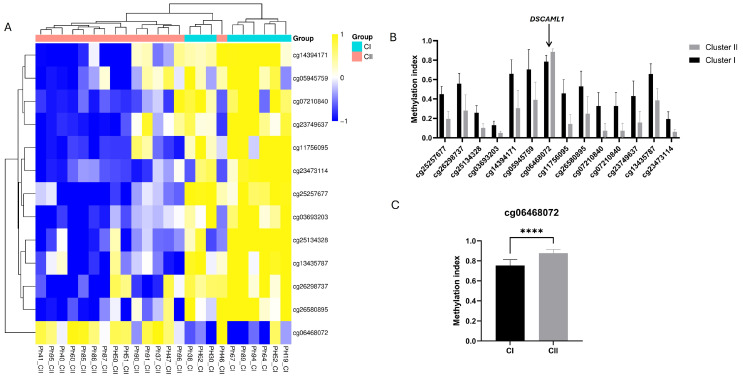
(**A**) Heatmap and hierarchical clustering of tumor samples based on DNA methylation profiles, stratified by genotype (Cluster I vs. Cluster II). Cyan indicates Cluster I and pink indicates Cluster II. All differentially methylated CpG sites for this comparison (FDR < 0.05, Δβ ≥ 0.2) are included. (**B**) Bar graph of differential DNA methylation between Cluster I and Cluster II. (**C**) Methylation index of CpG site cg06468072, located in the *DSCAML1* gene, across tumors from Cluster I and Cluster II. Statistical significance was determined by *t*-test; **** *p* = 0.0001.

Analysis of >850,000 CpG sites in 24 PPGLs, comparing 15 tumors from cluster II and 9 from cluster I, revealed 13 DMCs—(1) hypermethylated, (4) hypomethylated, and (8) partially methylated—in Cluster II (Figure 1A, Supplementary Table S2). No tumors corresponding to the rarer Cluster III (WNT pathway-driven) subtype were present in our cohort; therefore, all cluster-specific analyses were limited to Cluster I and Cluster II tumors.

These DMCs were annotated for genes such as *SLC39A14*, *SRP9*, *LINC01152*, *SKAP1*, *NXN*, *TCF12*, and *DSCAML1*. Most CpG sites exhibited partial methylation differences, indicating modest epigenetic variability between the clusters rather than broad methylation alterations across entire gene regions. The identified CpGs are associated with genes involved in diverse biological processes, including metal ion transport (*SLC39A14*), RNA processing (*SRP9*), intracellular signaling (*SKAP1*), oxidative stress regulation (*NXN*), and transcriptional regulation (*TCF12*). These discrete methylation changes likely represent fine-scale regulatory shifts contributing to the molecular heterogeneity observed between the PPGL clusters. Notably, cg06468072 associated with *DSCAML1* (DS Cell Adhesion Molecule-Like1) was hypermethylated in Cluster II (*p* = 0.0001) (Figure 1B,C). This CpG site is located in the gene body of *DSCAML1* and a member of the immunoglobulin superfamily (IgCAMs) cell adhesion molecules, and is situated in a CpG shore region.

Three additional CpG sites, cg16929692, cg24774961, and cg14474003, are located near cg06468072, forming a local CpG cluster. Among these, only cg24774961 showed a marginal difference between clusters (*p* = 0.048), while cg16929692 and cg14474003 were not significantly different, indicating that cg06468072 may be the primary driver of cluster-specific methylation in this region. An evaluation of genome-wide methylation patterns revealed that *DSCAML1* exhibited partial methylation in our cohort.

In comparison with TCGA data, *DSCAML1* shows a hypomethylated status in the 450K methylation data (Supplementary Figure S1). CpG site cg06468072, in the TCGA data, showed that the site is hypermethylated in both cluster samples, with no statistically significant difference between the two groups (*p* > 0.05), indicating a context-specific epigenetic variation rather than a universal oncogenic mechanism, but also likely due to greater ample heterogeneity and cohort composition.

**Figure 2:**
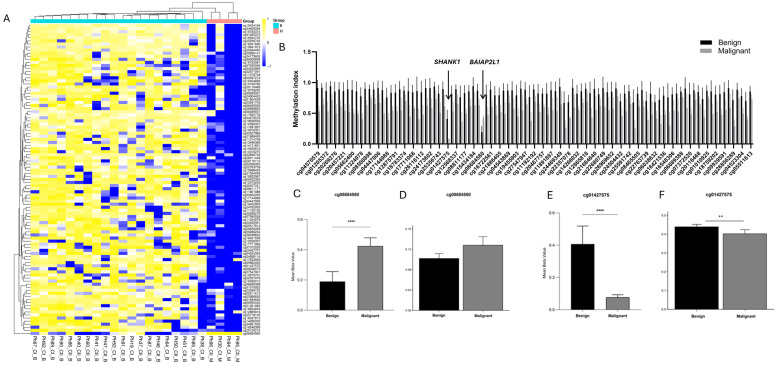
(**A**) Heatmap and hierarchical clustering of tumor samples based on DNA methylation profiles stratified by malignancy (benign vs. malignant). Cyan indicates benign and pink indicates malignant. All differentially methylated CpG sites for this comparison (FDR < 0.05, Δβ ≥ 0.2) are included. (**B**) Bar graph of differential DNA methylation between benign and malignant. (**C**) Methylation index of CpG site cg08684580, located in the *BAIAP2L1* gene, across tumors from benign and malignant clusters. (**D**) Methylation level of CpG site cg08684580 in the Illumina 450K array, comparing benign and malignant samples from TCGA data. (**E**) Methylation index of CpG site cg01427575, located in the *SHANK1* gene, across tumors from benign and malignant clusters. (**F**) Methylation level of CpG site cg01427575 comparing benign and malignant samples from TCGA data. Statistical significance was determined by *t*-test; ** *p* = 0.01 and **** *p* = 0.0001.

In the comparison between benign (*n* = 20) vs. malignant (*n* = 4) pheochromocytoma, 101 CpGs sites were differentially methylated, (17) hypermethylated, (1) hypomethylated and (83) partially methylated (Figure 2A, Supplementary Table S3).

Among these, CpG sites associated with *ZNF667*, *TEAD1*, *HDAC4*, *MSI2*, and *CCM2* were predominantly hypermethylated, while malignant tumors overall exhibited lower methylation levels compared to the benign group. These genes are known to participate in transcriptional regulation, chromatin remodeling, and the cellular signaling pathways that are frequently altered in PPGL. The observed hypomethylation may reflect epigenetic activation contributing to the enhanced proliferation, dedifferentiation, and vascular remodeling typical of malignant PPGLs, underscoring their potential role as epigenetically regulated drivers of tumor aggressiveness.

Notably, cg08684580 (*p* = 0.0008) was the only site not following this trend, being hypomethylated in malignant tumors (Figure 2B,C), the only hypomethylated site. The CpG site is located in the *BAIAP2L1* (BAI1-Associated Protein 2-Like 1) gene and encodes an adaptor protein also known as IRTKS (Insulin Receptor Tyrosine Kinase Substrate). The identified CpG site is located in the body-shore region of the *BAIAP2L1* gene on chromosome 7, in proximity to SNP rs555733464. CpG sites in gene body-shore regions are known to have regulatory potential, influencing gene expression and chromatin accessibility.

Two CpG sites near cg08684580, cg03252258 and cg15114304, are hypermethylated but did not show significant differences between benign and malignant tumors (*p* = 0.949 and *p* = 0.653, respectively), while the remaining CpGs in the region are partially methylated. The methylation landscape of *BAIAP2L1* differs between cohorts, with partial methylation observed in our samples. The fact that only cg08684580 is significantly hypomethylated in malignant tumors suggests that this site may represent a functionally distinct regulatory position within the CpG cluster, potentially playing a key role in tumor progression, whereas the neighboring sites may be less critical or more epigenetically stable.

The examination of TCGA 450K methylation data for CpG site cg08684580 revealed a hypomethylated profile across both benign and malignant tumor tissues, without a significant difference between groups (*p* > 0.05; Figure 2D). Similarly, *BAIAP2L1* as a whole shows predominantly hypomethylated patterns in TCGA, contrasting with the partial methylation observed in our samples, highlighting cohort-specific epigenetic variation and differences in CpG cluster regulation (Supplementary Figure S1).

Another interesting CpG site identified in our analysis was cg01427575, located within the *SHANK1* (SH3 and Multiple Ankyrin Repeat Domains 1) gene body (inside a CpG island), which encodes a postsynaptic scaffolding protein. In our cohort, this site displayed a marked differential methylation with a Δβ of 0.32, where the malignant group exhibited profound hypomethylation (β = 0.08) (*p* < 0.0001) (Figure 2E).

Two CpG sites surrounding cg01427575, namely cg12298955 and cg11573093, are hypermethylated but show no significant differences between malignant and benign tumors (*p* = 0.218 and *p* = 0.888, respectively). The remaining CpG sites in the region are partially methylated or hypomethylated, with no significant differences observed between the two groups. *SHANK1* gene showed partial methylation in both our cohort and the TCGA dataset (*p* = 0.013 and *p* > 0.05, respectively) (Supplementary Figure S1).

Similarly, analysis of TCGA data revealed that this cg08684580 is generally partially methylated in both benign and malignant tumors but shows a significantly lower methylation level in malignant tumors (*p* = 0.017), suggesting that reduced methylation at this locus may be a consistent feature of malignant pheochromocytomas and could hold potential as a biomarker for tumor aggressiveness (Figure 2F).

**Figure 3:**
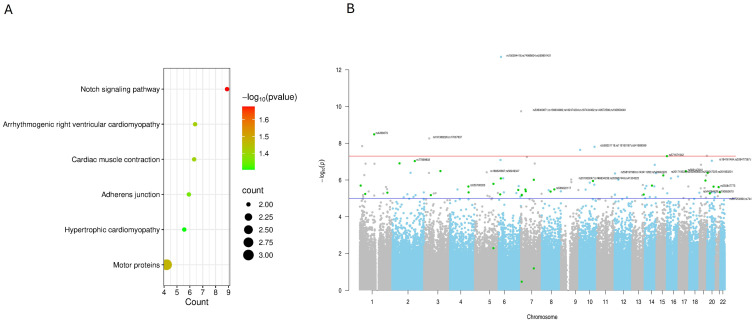
Differential pathway enrichment and genome-wide methylation associations between malignant and benign tumors: (**A**) bubble plot of enriched pathways (**B**) Manhattan plot of CpG associations (red: genome-wide significance; blue: suggestive threshold).

Due to the limited number of differentially methylated genes identified in the Cluster II versus Cluster I comparison after *p*-value correction (only three genes remaining), pathway enrichment analysis instead focused on the benign versus malignant comparison. The most significantly enriched pathways in this analysis included the Notch signaling pathway, motor proteins, cardiomyopathy-related pathways, and adherens junctions (Table 1) (Figure 3A).

Epigenome-wide association analysis was performed to identify CpG methylation differences between benign and malignant pheochromocytoma samples. The resulting Manhattan plot (Figure 3B) summarizes the distribution of association signals across all autosomal chromosomes, with the −log_10_(*p*-value) for each CpG site plotted against its genomic position. The red horizontal line indicates the genome-wide significance threshold (adjusted *p* < 0.05), while the blue line denotes suggestive significance. Multiple CpG sites surpassed the genome-wide threshold, indicating significant methylation differences between benign and malignant tumors. The most significant CpG sites were located on chromosome 6, forming a distinct cluster of highly associated loci (i.e., mapping *KIF13A*). In addition, a second notable cluster of six significant CpG sites was identified on chromosome 7, with all mapping around the *GET4* (Golgi to ER Transport Factor 4) gene.

**Figure 4:**
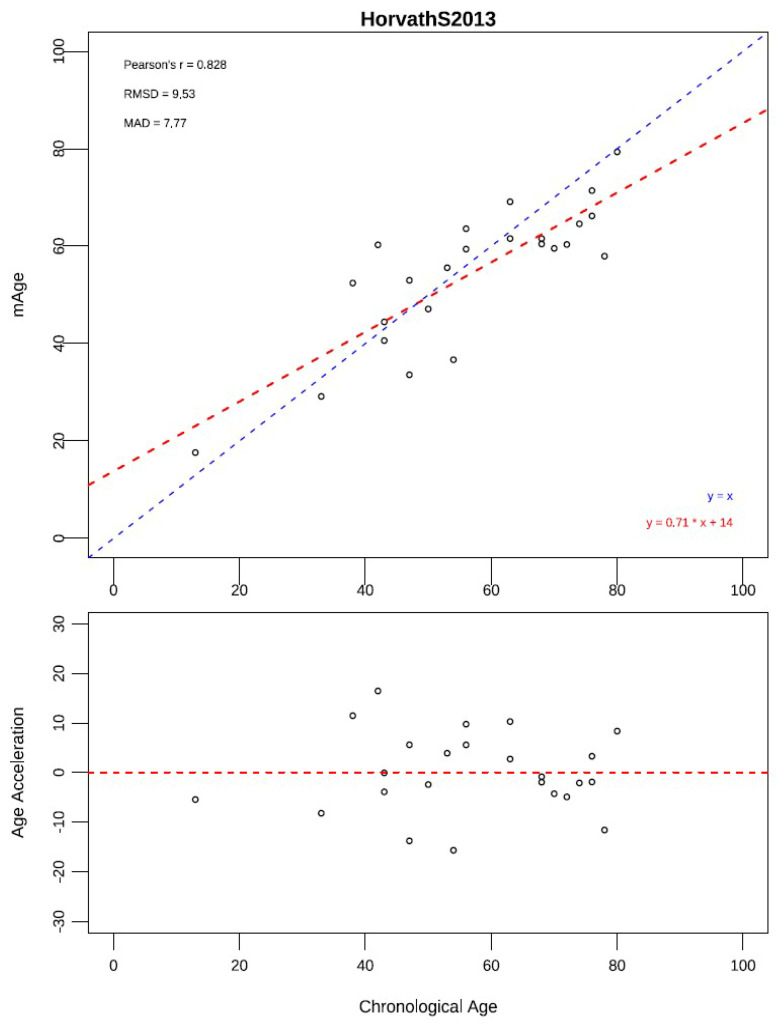
Correlation between methylation age and chronological age in PPGLs. Top panel: Methylation-derived age (mAge) plotted against chronological age, showing a strong positive correlation. The blue dashed line (y = x) represents the ideal one-to-one relationship, while the red dashed line (y = 0.71x + 14) shows the fitted linear regression (Pearson’s r = 0.828, slope = 0.71, intercept = 14). Root Mean Square Deviation (RMSD = 9.53) and Mean Absolute Deviation (MAD = 7.77) indicate moderate predictive accuracy. Bottom panel: Age acceleration (mAge—chronological age) versus chronological age, illustrating inter-tumoral variability in epigenetic aging. Points above the red dashed zero line denote accelerated epigenetic aging, while points below indicate deceleration.

Using the Horvath pan-tissue clock, methylation-derived age (mAge) strongly correlated with chronological age (Pearson *r* = 0.83). Age acceleration, defined as residual mAge after adjusting for chronological age, ranged from −15.7 to +16.5 years. Cluster I tumors exhibited significantly higher age acceleration (+3.7 years) than Cluster II (−2.5 years), whereas malignant tumors showed only a minor, non-significant trend toward positive acceleration (+0.73 vs. −0.31 years for benign) (Figure 4) (Supplementary Table S6).

### 3.2. Gene Expression Analysis

All included PPGL samples exhibited high RNA integrity numbers (RIN), with sample PH30 being excluded from the analysis. We investigated the gene expression profiles of these tumors to determine how they cluster relative to previously characterized PPGL genotypes, using hierarchical clustering of the current cohort.

**Figure 5:**
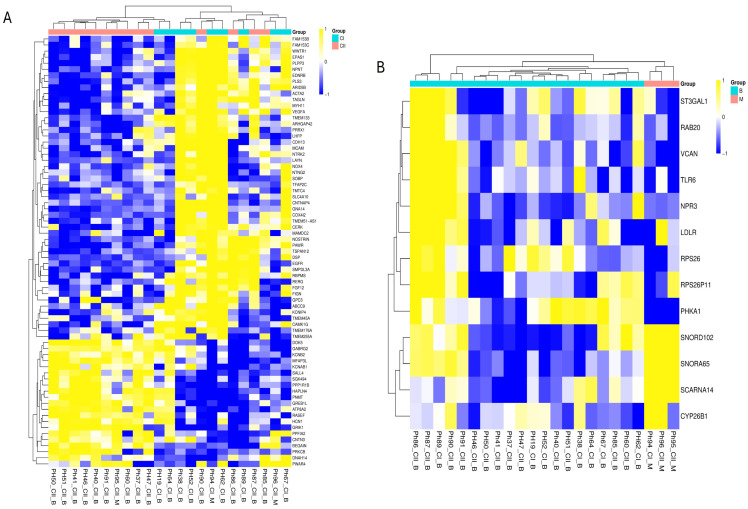
(**A**) Heatmap and hierarchical clustering of tumor samples based on gene expression profiles stratified by genotype (Cluster I vs. Cluster II). Cyan indicates Cluster I and pink indicates Cluster II. (**B**) Heatmap and hierarchical clustering of tumor samples based on gene expression profiles stratified by malignancy (benign vs. malignant). Cyan indicates benign and pink indicates malignant. Each heatmap includes all differentially expressed genes (DEGs) identified for the respective comparison using an q-value < 0.05 and an absolute fold change >2.

The hierarchical clustering of the microarray gene expression data did not yield a complete separation between the established Cluster I (CI) (pseudohypoxia-driven) and Cluster II (CII) (kinase/RTK-driven) pheochromocytomas, with samples from both groups intermixing along the dendrogram (Figure 5A). For the most variable genes (largest DEGs) represented here, transcriptional variation is influenced by factors beyond the canonical driver biology that defines these subtypes at the genomic and epigenomic levels (Supplementary Table S4). Nonetheless, certain subtype-related patterns are evident. Consistent with pseudohypoxia biology, *EPAS1* (*Endothelial PAS Domain Protein 1*, *encoding HIF-2α*) expression was higher in some CI cases, while adhesion and signaling molecules such as *EGFR* (*Epidermal Growth Factor Receptor*), *MCAM* (*Melanoma Cell Adhesion Molecule*, *CD146*), and *CDH13* (*Cadherin 13*, *H-cadherin*)—features of the mesenchymal/kinase-activated profile—were more prominent in subsets of CII tumors. Genes linked to cytoskeletal organization and adhesion (e.g., *DSP*, *ACTA2*, and *TAGLN*) showed heterogeneous patterns across both clusters, suggesting that malignant transformation and microenvironmental influences can blur subtype distinctions. Overall, while the heatmap confirms the presence of subtype-associated gene modules, it also underscores the complexity and partial overlap of transcriptional programs in pheochromocytoma, reinforcing the need for integrative analyses combining expression, methylation, and mutational data for robust molecular classification.

Differential expression analysis (DEG) identified 14 genes that were significantly altered between benign and malignant PPGL samples (Supplementary Table S5). Hierarchical clustering based on the expression of these genes revealed two major sample groups that largely corresponded to the benign (B) and malignant (M) phenotypes (Figure 5B). Malignant tumors predominantly clustered together and exhibited low expression of *RPS26*, *LDLR*, *ST3GAL1*, *PHKA1*, and *VCAN*, while showing higher expression of *SCARNA14*, *SNORA65*, and *CYP26B1*. The malignant PPGLs have a distinct transcriptional profile compared to benign tumors, characterized by the upregulation of small nucleolar RNAs and genes involved in metabolism and signaling that can be useful for diagnostic classification and identifying potential therapeutic targets. Among the differentially methylated genes, *DSCAML1* exhibited higher expression in cluster II compared to cluster I, although this difference did not reach statistical significance (FC = +2, *p* = 0.119). In contrast, *BAIAP2L1* showed significantly lower expression in the malignant group compared to the benign group (FC = −2.5, *p* = 0.017, FDR = 0.75), whereas *SHANK1* expression was similar in both malignant and benign group (FC = +1, *p* = 0.187).

### 3.3. Correlation Between Methylation and Gene Expression

Global correlation between methylation and expression was limited (Cluster I vs. II: r = −0.053, *p* = 0.90; benign vs. malignant: r = 0.17, *p* = 0.18) (Supplementary Figure S2A,B).

**Figure 6:**
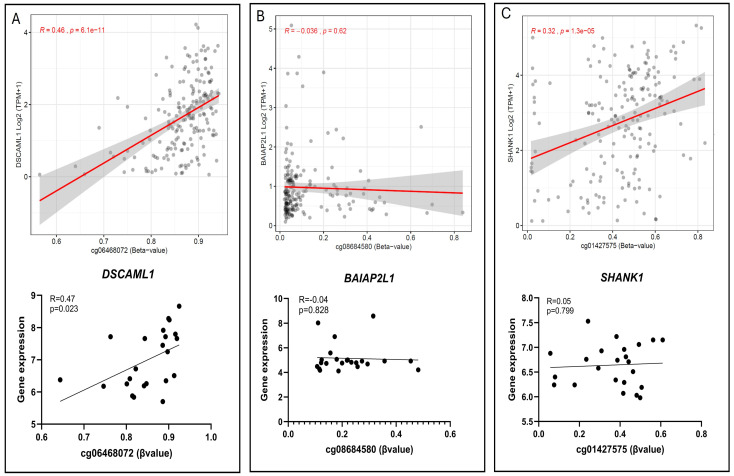
Correlation between DNA methylation (β-value) and gene expression for selected CpG sites: (**A**) cg12621303 and *DSCAML1*, (**B**) cg08684580 and *BAIAP2L1*, and (**C**) cg01427575 and *SHANK1*. Top panels: TCGA dataset (tumor samples, corresponding CpGs from the 450K array). Bottom panels: present study (tumor samples, CpGs from the 850K EPIC array).

At the gene level, *DSCAML1* revealed hypermethylation accompanied by elevated expression in cluster II compared to cluster I, with a positive correlation observed within our cohort (r = 0.47, *p* = 0.02). TCGA data for *DSCAML1* demonstrated a similar correlation (r = 0.46, *p* = 6 × 10^−11^) (Figure 6A). For *BAIAP2L1*, which was hypomethylated yet exhibited low expression in malignant and benign groups, no significant correlation was detected in our dataset (r = −0.04, *p* = 0.828) or in TCGA (r = −0.036, *p* = 0.62) (Figure 6B). Similarly, *SHANK1* was hypermethylated within the malignant group but did not show a significant correlation in our dataset (r = 0.05, *p* = 0.799). However, a significant correlation was detected in TCGA (r = 0.32, *p* = 1.3 × 10^−5^) (Figure 6C).

## 4. Discussion

In this study, we performed a comprehensive analysis of DNA methylation and gene expression in pheochromocytoma and paraganglioma (PPGL) samples, comparing both molecular clusters and benign versus malignant tumors. Using the Illumina Infinium EPIC (850K) array (EPIC array), we observed distinct methylation patterns that were largely concordant with known biology but also revealed novel, context-specific alterations.

Our integrative analysis of DNA methylation and gene expression in PPGL reveals complex, locus-specific epigenetic patterns that vary between molecular clusters and between benign and malignant tumors. A comparison of Cluster I and Cluster II tumors identified 13 differentially methylated CpG sites, most of which were hypomethylated or partially methylated in Cluster II. The exception, cg06468072 in *DSCAML1*, was hypermethylated in Cluster II and showed elevated expression in our cohort, suggesting that gene body methylation in CpG shore regions may enhance transcription rather than suppress it. TCGA 450K data indicated that this locus is hypermethylated across both CI and CII tumors, without significant differences, suggesting that the hypermethylation observed in Cluster II likely reflects secondary or context-dependent epigenetic variation rather than an oncogenic mechanism. The CpG island methylator phenotype (CIMP) characteristic of SDH-mutant, pseudohypoxic Cluster I tumors [3] is not recapitulated in the kinase-signaling Cluster II subgroup, which generally exhibits methylation levels close to normal chromaffin tissue [13,16]. Moreover, isolated hypermethylation events in Cluster II rarely coincide with recurrent promoter silencing or the transcriptional repression of canonical cancer genes, supporting their interpretation as likely context-dependent epigenetic alterations rather than driver events [17,18].

In the comparison between benign and malignant tumors, a larger number of methylation differences were observed, with 101 CpG sites reaching statistical significance. Malignant tumors exhibited global hypomethylation compared to benign tumors, except for specific loci such as cg08684580 in *BAIAP2L1*, which showed higher methylation levels in malignant samples. Although pseudohypoxic tumors are often associated with a hypermethylator phenotype, driven by the oncometabolite-induced inhibition of DNA demethylases and widespread CpG island methylation, more recent methylome-wide investigations have demonstrated that DNA methylation patterns in PPGL are highly dependent on molecular subtype and driver mutation, rather than malignant behavior on its own [3,19]. For example, a genome-wide DNA methylation study of 39 PPGLs identified two distinct methylation clusters: one hypermethylated (mostly SDHx-mutated) and one hypomethylated and, crucially, malignant tumors were distributed in the hypomethylated cluster, with a higher burden of chromosomal instability and copy number aberrations compared with benign tumors [20].

A recent multi-omics study further confirmed that metastatic PPGLs often exhibit intermediate methylation states (neither fully hypermethylated nor globally hypomethylated), including at promoter and gene-body CpGs, arguing against a uniform global hypermethylation leading to malignancy [13]. These findings indicate that epigenetic reprogramming in PPGL is context- and locus-specific, variable across subtypes, and likely influenced by genetic background, tumor cell origin, and microenvironment factors, although the functional consequences require further experimental validation [21]. 

These findings suggest that epigenetic changes in malignant PPGL are context-specific, locus-dependent, and influenced by both genetic and microenvironmental factors, explaining why only certain CpGs, such as cg08684580, show increased methylation in malignant tumors.

The inverse *BAIAP2L1* methylation–expression relationship suggests potential regulatory silencing of *BAIAP2L1*, consistent with the broader role in cancer cell migration and metastasis [22]. Conversely, the additional hypomethylation at *SHANK1* may represent secondary chromatin relaxation accompanying malignant progression, as broad genomic hypomethylation has been linked to chromosomal instability and dedifferentiation [23]. Taken together, these data indicate that malignant PPGLs display a mixed methylation landscape, dominated by global hypomethylation but punctuated by locus-specific hypermethylation events, as in *BAIAP2L1*, *which* may reflect selective, tumor-specific regulatory adaptation rather than a uniform epigenetic trend, though experimental validation is needed to confirm functional effects.

Pathway enrichment of differentially methylated genes further revealed significant involvement of Notch signaling, adherens junctions, motor proteins, and cardiomyopathy-related pathways, suggesting the potential involvement of epigenetically regulated genes in cell adhesion, signaling, and cytoskeletal dynamics [24,25].

Notably, Notch pathway components, including receptors, ligands, and downstream effectors such as HES and HEY, are expressed across major PPGL cellular compartments, including neuroendocrine chief cells, sustentacular glial-like cells, and endothelial cells [26]. The activation of Notch1 requires proteolytic release of the intracellular domain and co-activators such as MAML, which are functionally connected to transcriptional programs similar to those observed in Cluster III expression patterns. Previous studies indicate that Notch signaling can be modulated via epigenetic mechanisms and microRNA-mediated regulation (e.g., miR-200 and miR-34 families) even in the absence of genetic alterations [26]. While direct evidence of canonical Notch activation (NICD cleavage, target gene induction) in PPGL is limited, our methylation data suggest potential dysregulation of the pathway, which may influence cellular plasticity, differentiation, and intercellular communication in malignant tumors. These findings provide a rationale for future investigations using integrated transcriptomic, proteomic, and functional assays to determine whether methylation changes correspond to Notch activation and contribute to malignant phenotypes [2,27].

Epigenome-wide association analysis identified clusters of CpGs on chromosomes 6 and 7, mapping to genes such as *KIF13A* and *GET4*, reinforcing the concept that methylation changes in cytoskeleton- and transport-related genes could contribute to tumor progression. The dysregulation of kinesin family members, for example, has been implicated in chromosomal instability, altered intracellular trafficking, and metastatic behavior in solid tumors [28,29]. Collectively, these data suggest a model in which malignant PPGLs undergo selective epigenetic rewiring, combining global hypomethylation with locus-specific hyper- or hypomethylation events that target functional pathways critical to invasion, adhesion, and cytoskeletal organization.

Methylation-derived age was estimated using the Horvath pan-tissue clock, a validated algorithm that calculates biological age from 353 CpG sites across multiple tissues and captures tumor-associated epigenetic dysregulation [15]. In our cohort, Cluster I tumors exhibited a positive age acceleration (+3.66 years), whereas Cluster II tumors showed negative acceleration (−2.53 years), showing that epigenetic over-aging is primarily associated with the pseudohypoxic molecular subtype rather than kinase-signaling tumors. In contrast, malignant tumors showed only a modest, non-significant trend toward higher age acceleration (+0.73 vs. −0.31 years in benign tumors). These findings suggest that methylation age acceleration in PPGL is driven more strongly by molecular subtype than by malignancy. Previous research using epigenetic clocks in large, population-based cohorts has demonstrated associations between age acceleration and cancer risk or outcomes, but these associations tend to be modest and are often reported in studies with substantially larger sample sizes, indicating the need for replication in larger and independent PPGL cohorts to clarify the potential relevance of age acceleration as a marker of malignancy [15].

Gene expression profiling revealed a partial overlap between Cluster I and II tumors, underscoring that transcriptional heterogeneity in PPGLs is shaped not only by canonical driver mutations but also by epigenetic regulation and microenvironmental influences [9,13]. Subtype-specific expression patterns emerged: *EPAS1* was elevated in Cluster I tumors, consistent with the pseudohypoxic/SDH-mutant phenotype, while subsets of Cluster II tumors showed higher expression of adhesion and signaling molecules such as *EGFR*, *MCAM* and *CDH13*, suggesting the selective activation of pathways involved in cell–cell communication, motility and growth-factor signaling [24,25].

Differential expression analysis between benign and malignant tumors identified 14 significantly altered genes, including the downregulation of *RPS26*, *LDLR*, *ST3GAL1*, *PHKA1* and *VCAN*, and upregulation of small nucleolar RNAs (*SCARNA14*, *SNORA65*) and *CYP26B1*. These changes implicate RNA processing, metabolism, and signaling pathways in malignant progression, highlighting potential biomarkers or therapeutic targets [30,31].

Integration with methylation data revealed gene-specific epigenetic effects. Among differentially methylated genes, *DSCAML1* exhibited higher expression in Cluster II tumors (not statistically significant), whereas *BAIAP2L1* showed significantly lower expression in malignant tumors, consistent with locus-specific hypermethylation discussed previously. In contrast, *SHANK1* expression remained largely unchanged despite hypomethylation, underscoring that methylation changes do not uniformly predict transcriptional output, and that additional regulatory layers such as chromatin context and transcription-factor occupancy likely contribute to the observed expression patterns [17,32].

These results illustrate that malignant PPGLs are characterized by a complex interplay between transcriptional programs and epigenetic modifications, with selective gene-expression changes in adhesion, signaling, and RNA-processing pathways that may inform prognostic assessment and therapeutic strategies.

Correlation analysis between methylation and gene expression revealed limited global associations, with no significant correlations across Cluster I versus II tumors or between benign and malignant groups, highlighting that DNA methylation alone does not uniformly dictate transcriptional output in PPGLs [9,13]. Overall, while the correlations between methylation and expression in our cohort were generally weaker and often did not reach statistical significance, the direction of effects largely mirrored trends observed in TCGA datasets, suggesting the consistency of locus-specific regulatory patterns across two independent cohorts. At the gene-specific level, *DSCAML1* exhibited a positive correlation between methylation and expression in our cohort, in contrast to the negative correlation observed in TCGA data, while *BAIAP2L1* and *SHANK1* showed limited or cohort-dependent correlations. These differences suggest that the locus-specific methylation effects are context-dependent, influenced by chromatin state, transcription factor occupancy, three-dimensional genome organization, or post-transcriptional regulation [33,34]. Collectively, these findings suggest that integrative analyses combining methylation, transcriptional profiling, and chromatin context are required to fully understand gene regulation in PPGLs, and that methylation may function as one component of a multilayered regulatory network rather than a primary driver of expression changes.

Overall, our results demonstrate that PPGLs exhibit cluster- and malignancy-specific methylation alterations, with malignant tumors showing more extensive global hypomethylation alongside distinct transcriptional profiles. While global methylation changes alone do not reliably predict gene expression, specific loci such as *DSCAML1*, *BAIAP2L1*, and *SHANK1* display context-dependent regulatory patterns that may modulate tumor biology in a locus- and cohort-specific manner, reflecting a complex interplay between epigenetic modification, chromatin structure, and transcription factor occupancy [13]. The pathway-level enrichment and genome-wide clustering of differentially methylated loci are suggested to have functional consequences in cell adhesion, cytoskeletal organization, intracellular transport, and signaling, consistent with previous observations linking epigenetic remodeling to structural and signaling network alterations in malignant PPGLs [25].

## 5. Conclusions

In summary, our integrative analysis of DNA methylation and gene expression reveals that PPGLs exhibit cluster- and malignancy-specific epigenetic alterations, with malignant tumors displaying changes in pathways governing Notch signaling, cell adhesion, cytoskeletal organization, and RNA processing. While global methylation changes do not consistently predict transcriptional output, locus-specific effects, notably in *DSCAML1*, *BAIAP2L1*, and *SHANK1*, suggest potential mechanistic relevance and potential functional impact on tumor biology. Methylation age analysis indicated modest age acceleration in pseudohypoxic Cluster I tumors, suggesting subtype-specific epigenetic remodeling. These findings enhance our understanding of PPGL molecular heterogeneity and provide a foundation for molecular classification, biomarker discovery, and the identification of candidate therapeutic targets. The limitations of this study include a relatively small cohort and weak correlations between global methylation and gene expression, highlighting the need for larger, multi-center studies with single-cell resolution and functional validation. Future work should focus on experimentally dissecting the roles of key epigenetically regulated genes to clarify their contributions to tumor progression and therapeutic vulnerability.

## Figures and Tables

**Table 1:** Differential pathway enrichment in malignant and benign tumors.

ID	Description	Gene Ratio	Bg Ratio	Rich Factor	Fold Enrichment	*p*-Value
hsa04330	Notch signaling pathway	2/31	62/8541	0.032	8.888	0.021
hsa04814	Motor proteins	3/31	197/8541	0.015	4.196	0.033
hsa05412	Arrhythmogenic right ventricular cardiomyopathy	2/31	86/8541	0.023	6.407	0.038
hsa04260	Cardiac muscle contraction	2/31	87/8541	0.023	6.334	0.039
hsa04520	Adherens junction	2/31	93/8541	0.022	5.925	0.045
hsa05410	Hypertrophic cardiomyopathy	2/31	99/8541	0.020	5.565	0.049

## Data Availability

Data supporting the reported results are available upon reasonable request from the corresponding author.

## Supplementary Materials

The following supporting information can be downloaded at: https://www.mdpi.com/article/10.3390/cells15020198/s1, Table S1: Cohort characteristics; Table S2: Differentially Expressed Methylation (DEM) sites between Cluster I and Cluster II in the PPGL cohort; Table S3: Differentially Expressed Methylation (DEM) sites between benign and malignant tumors in the PPGL cohort; Table S4: Differentially Expressed Genes (DEG) sites between Cluster I and Cluster II in the PPGL cohort; Table S5: Differentially Expressed Genes (DEG) sites between benign and malignant tumors in the PPGL cohort; Table S6: DNA methylation-derived age and age acceleration in PPGL samples estimated using the Horvath pan-tissue clock; Figure S1: Gene methylation status: (A) DSCAML1 (B) *BAIAP2L1*, and (C) *SHANK1*. Top panels: TCGA dataset (tumor samples, corresponding CpGs from the 450K array). Bottom panels: present study (tumor samples, CpGs from the 850K EPIC array); Figure S2: Correlation between gene expression and DNA methylation indices in: (A) Cluster II vs. Cluster I Tumors and (B) benign vs. malignant tumors.

## Abbreviations

The following abbreviations are used in this manuscript; SDHA (Succinate Dehydrogenase Complex Flavoprotein Subunit A); SDHB (Succinate Dehydrogenase Complex Iron-Sulfur Subunit B); SDHC (Succinate Dehydrogenase Complex Subunit C); SDHD (Succinate Dehydrogenase Complex Subunit D); SDHx (general abbreviation for SDH complex genes); VHL (Von Hippel–Lindau Tumor Suppressor); RET (REarranged during Transfection proto-oncogene, Receptor Tyrosine Kinase); NF1 (Neurofibromin 1); TMEM127 (Transmembrane Protein 127); MAX (MYC Associated Factor X); EPAS1 (Endothelial PAS Domain Protein 1, HIF-2α); ATRX (Alpha Thalassemia/Intellectual Disability Syndrome X-Linked); FH (Fumarate Hydratase); MAML3 (Mastermind Like Transcriptional Coactivator 3); TET (Ten-Eleven Translocation, DNA demethylase family); DSCAML1 (Down Syndrome Cell Adhesion Molecule-Like 1); SLC39A14 (Solute Carrier Family 39 Member 14); SRP9 (Signal Recognition Particle 9 kDa); LINC01152 (Long Intergenic Non-Protein Coding RNA 1152); SKAP1 (Src Kinase Associated Phosphoprotein 1); NXN (Nucleoredoxin); TCF12 (Transcription Factor 12); ZNF667 (Zinc Finger Protein 667); TEAD1 (TEA Domain Transcription Factor 1); HDAC4 (Histone Deacetylase 4); MSI2 (Musashi RNA Binding Protein 2); CCM2 (Cerebral Cavernous Malformations 2); BAIAP2L1 (BAI1-Associated Protein 2-Like 1, also IRTKS); SHANK1 (SH3 and Multiple Ankyrin Repeat Domains 1); EGFR (Epidermal Growth Factor Receptor); MCAM (Melanoma Cell Adhesion Molecule, CD146); CDH13 (Cadherin 13, H-cadherin); DSP (Desmoplakin); ACTA2 (Actin Alpha 2, Smooth Muscle); TAGLN (Transgelin); RPS26 (Ribosomal Protein S26); LDLR (Low-Density Lipoprotein Receptor); ST3GAL1 (ST3 Beta-Galactoside Alpha-2,3-Sialyltransferase 1); PHKA1 (Phosphorylase Kinase Regulatory Subunit Alpha 1); VCAN (Versican); SCARNA14 (Small Cajal Body-Specific RNA 14); SNORA65 (Small Nucleolar RNA, H/ACA Box 65); CYP26B1 (Cytochrome P450 Family 26 Subfamily B Member 1); KIF13A (Kinesin Family Member 13A); GET4 (Golgi to ER Transport Factor 4); HES (Hairy and Enhancer of Split, Notch pathway transcription factor); HEY (Hes-Related Family bHLH Transcription Factor with YRPW Motif, Notch pathway).
