# Supplementary figures and images for: Integrative Epigenomic and Transcriptomic Profiling Define Malignancy- and Cluster-Specific Signatures in Pheochromocytomas and Paragangliomas
Source: Cells. 2026 Jan 20;15(2):198. doi: 10.3390/cells15020198 (PMC12839883; doi:10.3390/cells15020198)

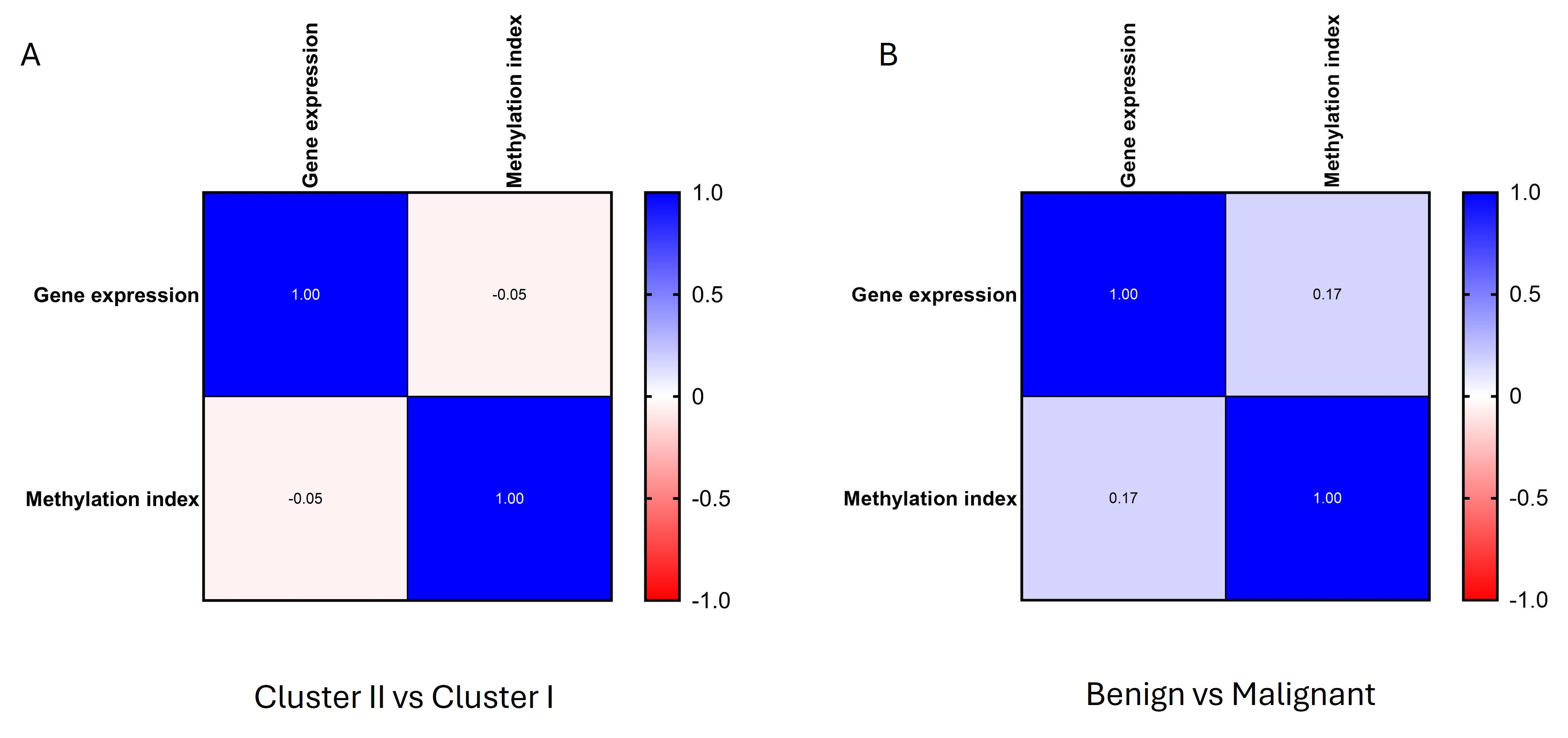

Supplement: Supplementary file 1 [file cells-15-00198-s001.zip › cells-4086896-supplementary/Suplementary Figure S2_vf.jpg]

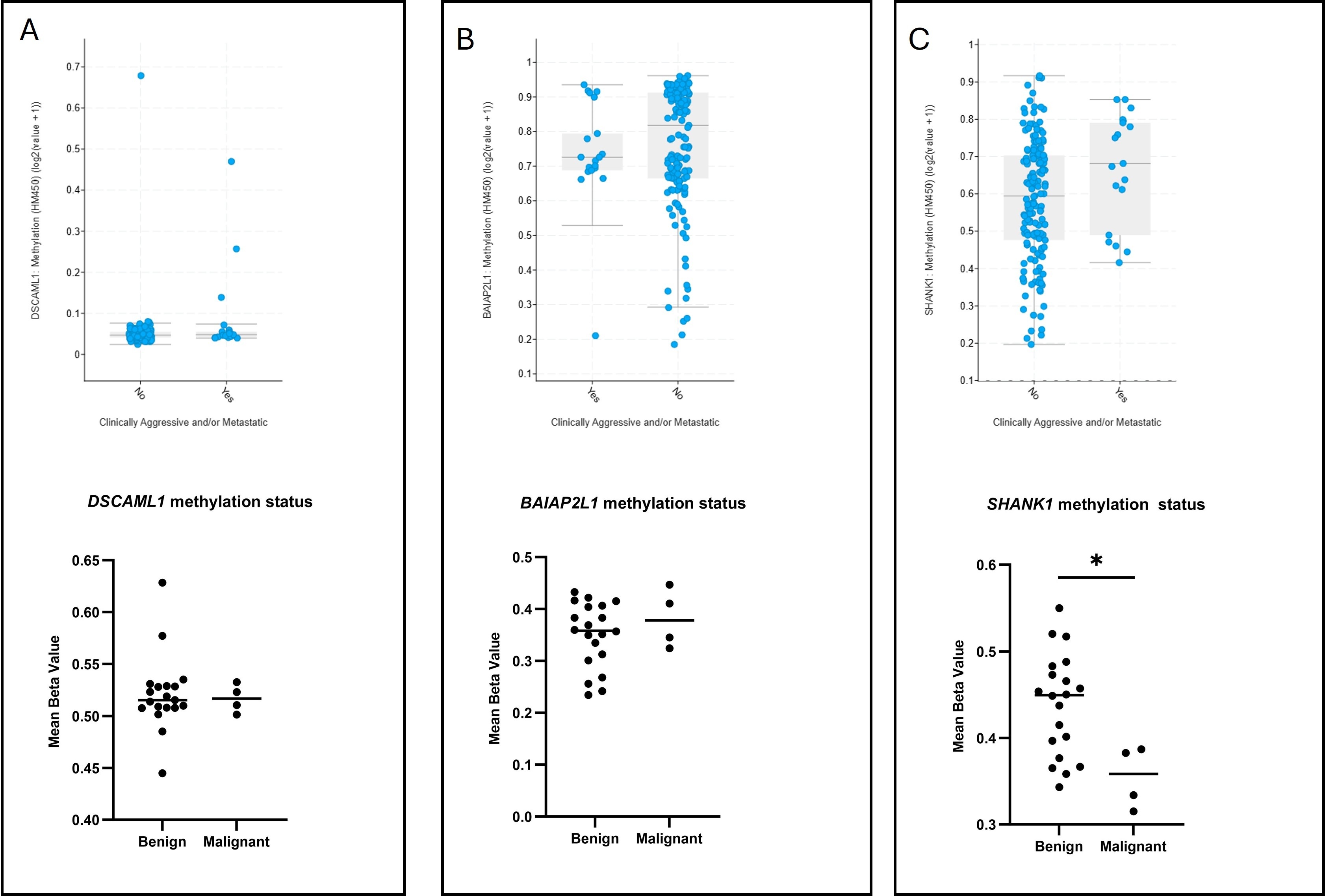

Supplement: Supplementary file 1 [file cells-15-00198-s001.zip › cells-4086896-supplementary/Supplemnetary Figure S1_Adj.jpg]
